# Supplementary material for: p50 mono-ubiquitination and interaction with BARD1 regulates cell cycle progression and maintains genome stability
Source: Nat Commun. 2020 Oct 6;11:5007. doi: 10.1038/s41467-020-18838-2 (PMC7538584; doi:10.1038/s41467-020-18838-2)
Supplement: Supplementary file 1 — Supplementary Information [file 41467_2020_18838_MOESM1_ESM.pdf]

SUPPLEMENTARY INFORMATION

p50 mono-ubiquitination and interaction with BARD1 regulates cell cycle progression and maintains genome stability

Longtao Wu, Clayton D. Crawley, Andrea Garofalo, Jackie W. Nichols, Paige-Ashley Campbell, Galina F. Khramtsova, Olufunmilayo I. Olopade, Ralph R. Weichselbaum and Bakhtiar Yamini.

Supplementary Figures

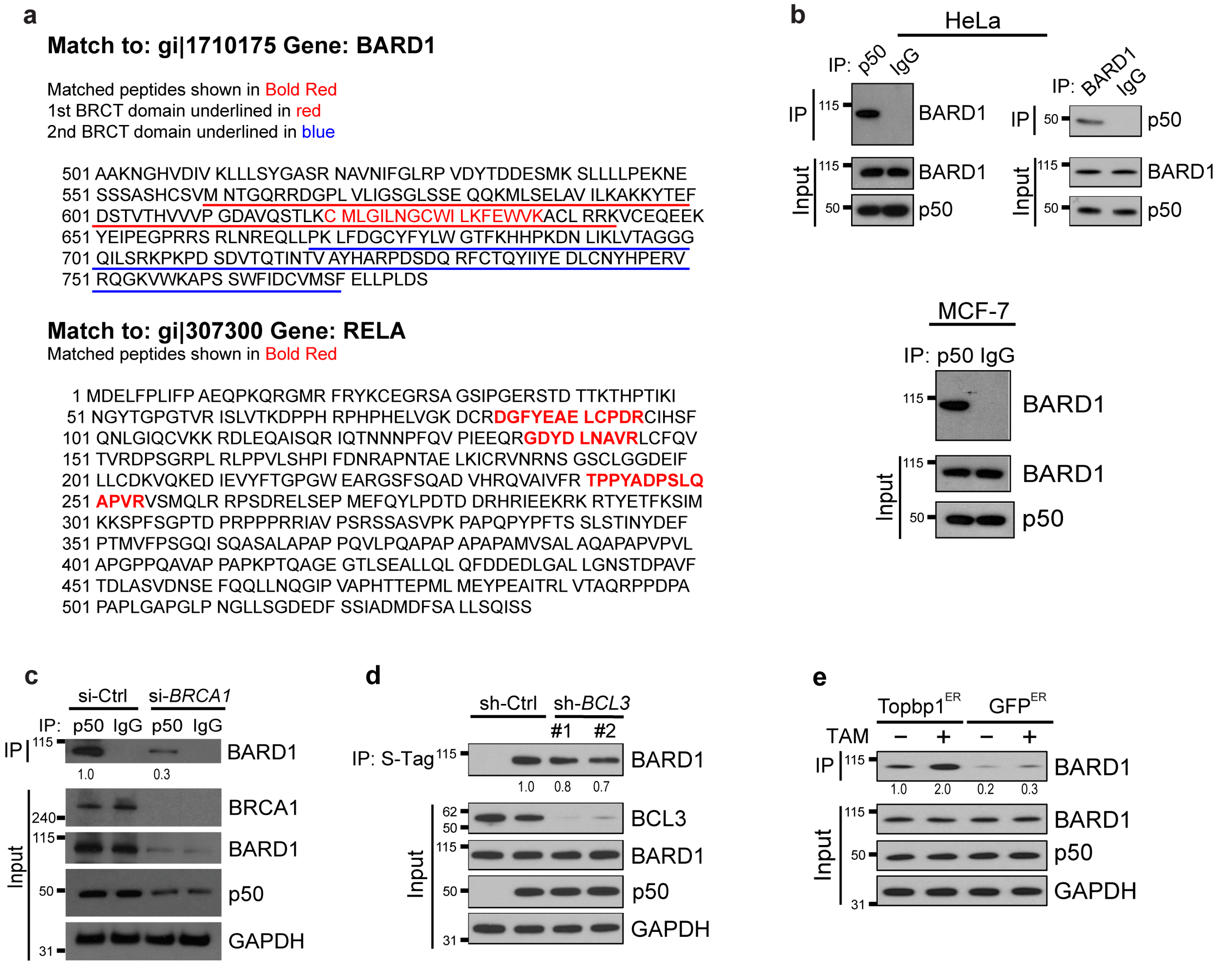

Supplementary Fig. 1 p50 binds the BARD1 BRCT domains. (**a**) Sequences of BARD1 and RELA peptides interacting with p50 identified by MS/MS. (**b**) Co-IP using HeLa (upper) and MCF-7 (lower) cell extract following endogenous protein IP and IB with the indicated antibodies. (**c**) Co-IP in U87 cells transfected with siRNA SMART pool targeting *BRCA1* or control sequence. IP was then performed with anti-p50 or IgG control and IB with anti-BARD1 antibody. Input sample was analyzed with the antibodies indicated. (**d**) Co-IP in 293T cells infected with two independent lentiviral vectors expressing sh-RNA targeting *BCL3* or a scrambled sequence. Cells were transfected with S-p50^{wt} and IP performed with S-agarose and IB with anti-BARD1. GAPDH was used as loading control. (**e**) HEK293T cells expressing TopBP1^{ER} or GFP^{ER} were transfected with S-tag p50 and treated with TAM (4 hrs). IP with S-agarose and IB with anti-BARD1. All blots are representative of at least two biologically independent experiments. Analysis of fold-change normalized to control lane shown below IB where indicated.

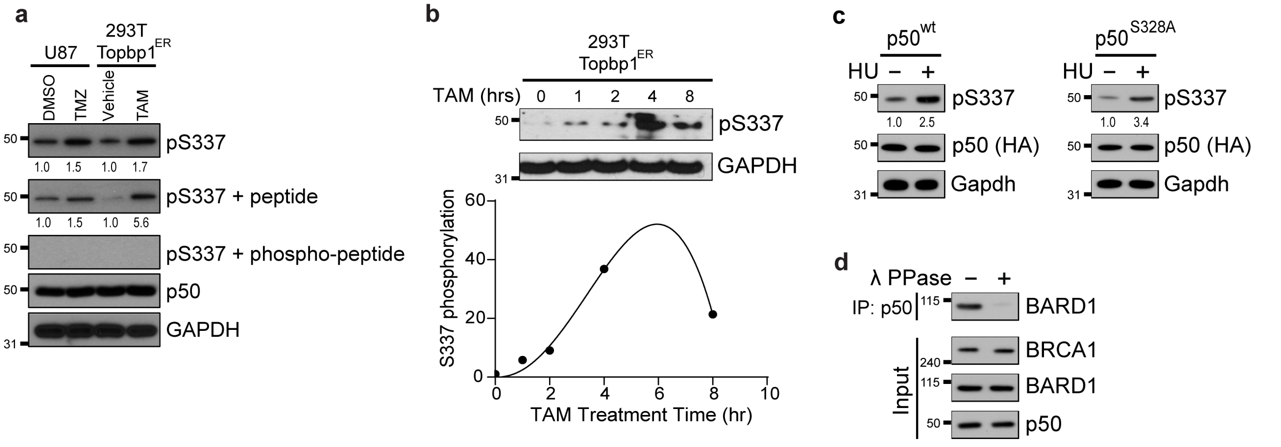

Supplementary Fig. 2 ATR induces p50 S337 phosphorylation. (**a**) Immunoblot (IB) in U87 cells treated with DMSO or temozolomide (TMZ, 100 μM for 16 hrs) or 293T cells stably expressing TopBP1^{ER} treated with vehicle or 4-hydroxy-tamoxifen (TAM, 500 nM for 4 hrs). IB with anti-phospho-p50-S337 (pS337) antibody either alone or following pre-incubation with S337 peptide (Sequence: QLRRKSDLETSEPKC) or S337 phospho-peptide (Sequence: QLRRKpSDLETSEPKC) at 1:10 ratio. Anti-p50 and anti-GAPDH were used as loading controls. (**b**) IB (upper) in 293T cells stably expressing TopBP1^{ER} treated with vehicle or TAM (500 nM) for the indicated times. IB with anti-phospho-p50-S337 (pS337) antibody. Quantification (lower) of relative p50 S337 phosphorylation at the indicated time. (**c**) IB in *Nfkb1$^{-/-}$* MEFs transfected with p50^{wt} (left) or p50^{S328A} (right) treated with vehicle or HU (2 mM, 4 hrs) probed with anti-pS337 antibody. (**d**) Co-IP in 293T cells following IP with anti-p50 and IB with the indicated antibody. Lysates were treated with lambda phosphatase (λ PPase) or vehicle as indicated. All blots are representative of at least two biologically independent experiments. Analysis of fold-change normalized to control lane shown below IB where indicated.

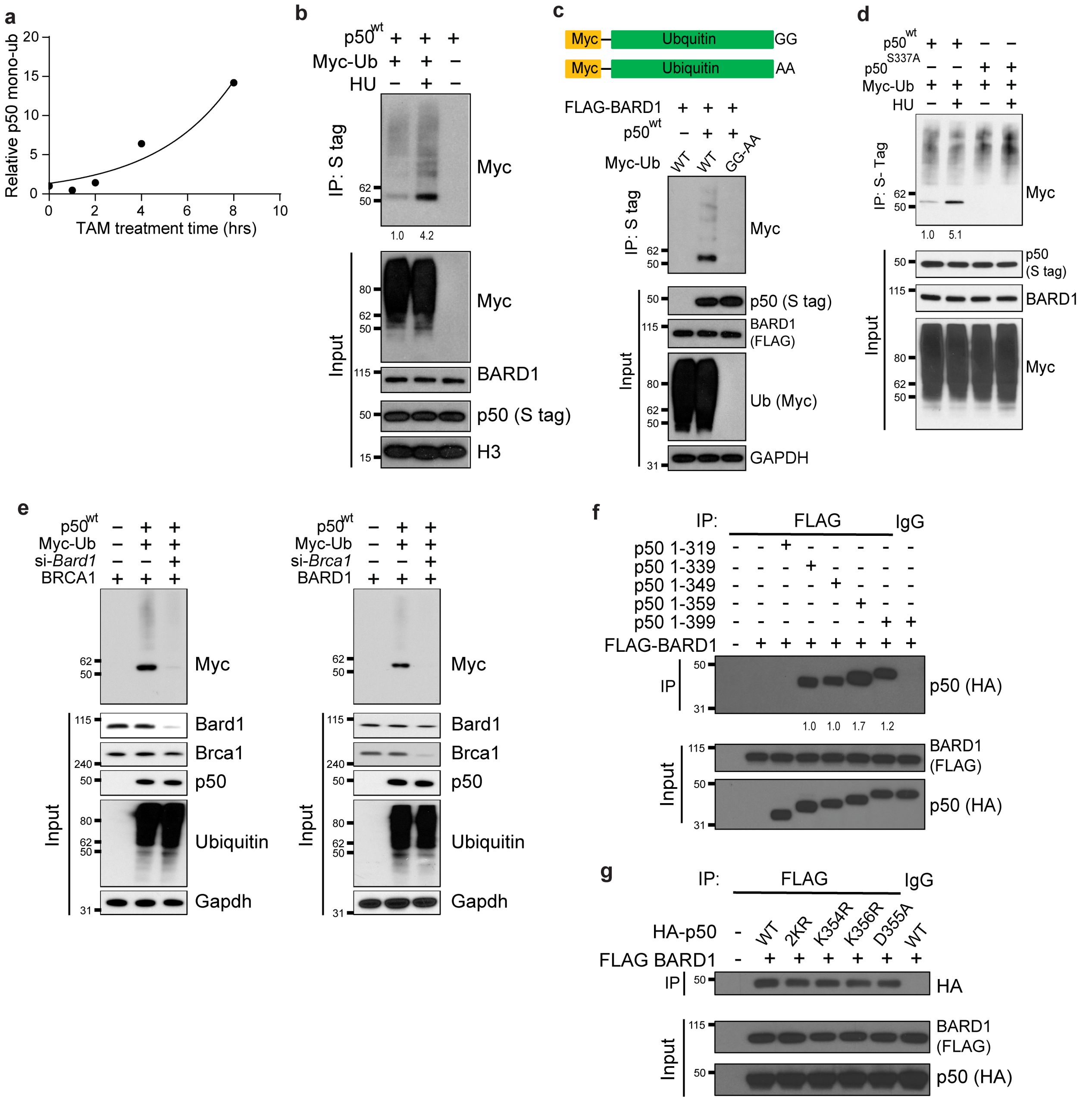

Supplementary Fig. 3 BARD1 binding promotes p50 mono-ubiquitination. (**a**) Quantification of Fig. 3a, demonstrates relative amount of mono-ubiquitinated p50 at the indicated time following TAM treatment. (**b**) Immunoblot (IB) in nuclear extract from 293T cells transfected with empty vector or Myc-Ubiquitin (Myc-Ub) and S-tag p50^{wt} treated with hydroxyurea (HU, 2 mM for 4 hrs). IP of nuclear extract was performed with S-agarose and IB with anti-Myc antibody. Input nuclear sample was probed as indicated. (**c**) 293T cells were transfected with FLAG-BARD1, S-p50^{wt} or empty vector and Myc-tagged wild type or mutant (G75A/G76A, GG-AA) ubiquitin (Myc-Ub). IP was performed with S-agarose and IB with anti-Myc antibody. Inputs were probed with antibody against

the indicated tag. Inset: schematic of Myc-tagged wild type (GG) and mutant (AA) ubiquitin constructs. (**d**) 293T cells transfected with Myc-Ub, S-tag p50^{wt} or p50^{S337A} and treated with HU (2 mM, 4 hrs). IP with S-agarose and IB with anti-Myc antibody. (**e**) IB in *Nfkb1*$^{-/-}$ MEFs expressing p50^{wt}, BARD1 and BRCA1 and either si-*Bard1* (left) or si-*Brca1* (right). IP with S-agarose and IB as indicated. (**f**) Co-IP in 293T cells transfected with FLAG-BARD1 and HA-tagged p50 C-terminal deletion constructs containing the indicated amino acids. IP was performed with anti-FLAG antibody and IB with anti-HA antibody. (**g**) Co-IP in 293T cells transfected with FLAG-BARD1 and HA-p50 wild type and mutants as indicated. IP was performed with anti-FLAG antibody and IB with anti-HA antibody. All blots are representative of at least two biologically independent experiments. Analysis of fold-change normalized to control lane shown below IB where indicated.

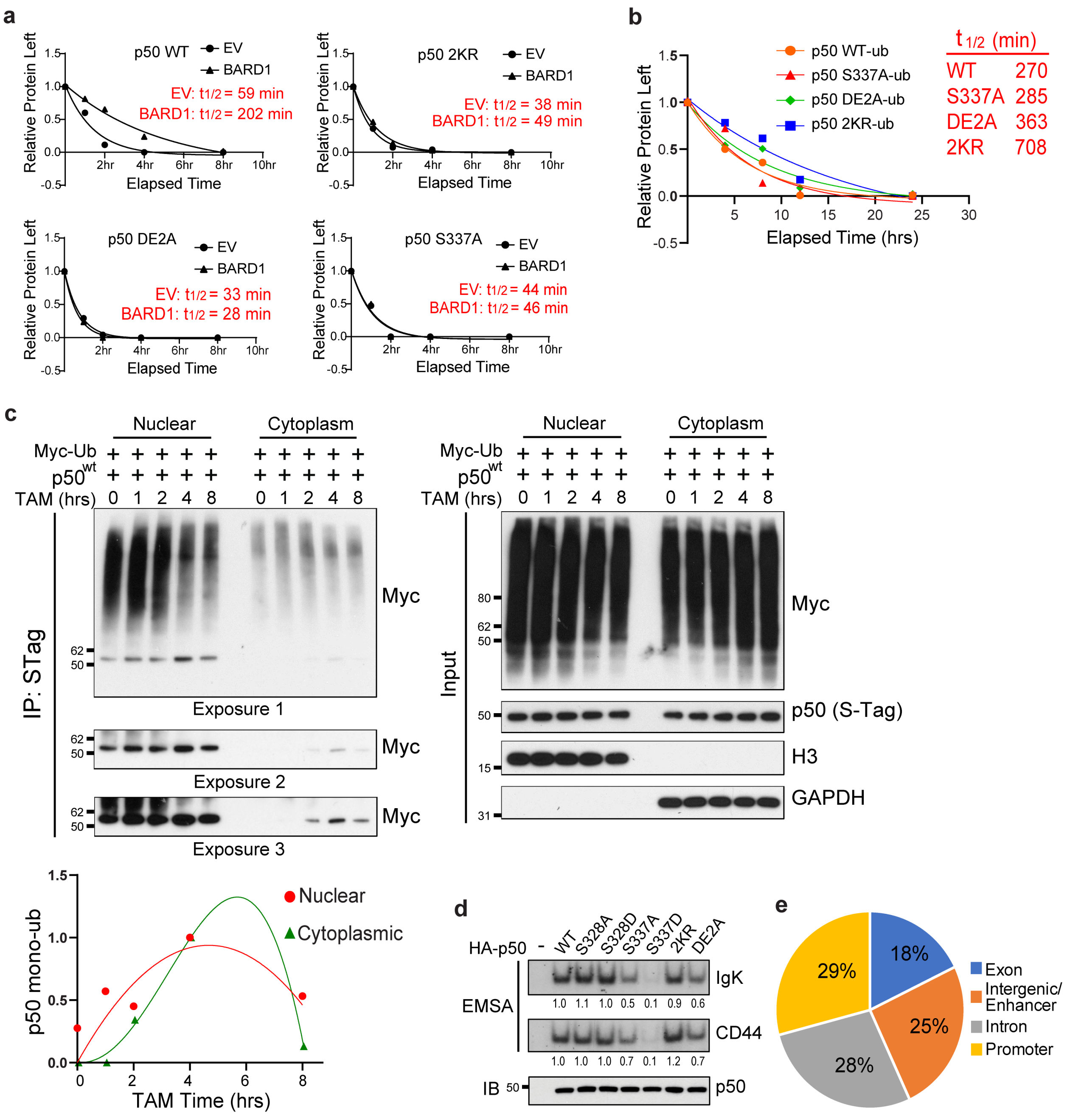

Supplementary Fig. 4. Mono-ubiquitination modulates p50 chromatin recruitment. (**a**) Quantification of Fig. 5a indicates relative amount of indicated p50 protein remaining at each time point in the presence of BARD1 or empty vector (EV). Half-life ($t_{1/2}$) in the presence of BARD1 or EV indicated in red. (**b**) Quantification of Fig. 5c indicates relative amount of indicated p50-Ub fusion protein remaining at each time point. $t_{1/2}$ of each protein is indicated in red. Compare data with Supplementary Fig. 4a. (**c**) 293T cells expressing TopBP1^{ER} were transfected with Myc-Ub, S-p50^{wt} and treated with TAM for the indicated time and then fractionated into nuclear and cytoplasmic

compartments. IP (left) was performed with S-agarose and IB with anti-Myc antibody. Input (right) was probed with the indicated antibodies. H3 and GAPDH were used as loading control for the corresponding cell fraction. Lower plot demonstrates quantification of relative amount of mono-ubiquitinated p50 in the nucleus and cytoplasm normalized to the peak value (4 hrs). (**d**) EMSA (upper) in 293T cells transfected with HA-tagged p50 constructs using the oligonucleotide probe from the indicated gene. Membranes were analyzed by autoradiography (upper) and IB (lower) with anti-HA antibody to demonstrate equal construct expression. (**e**) Pie chart demonstrating the distribution of p50 Chip-Seq peaks annotated to specific genomics features. All blots are representative of at least two biologically independent experiments. Analysis of fold-change normalized to control lane shown below IB where indicated.

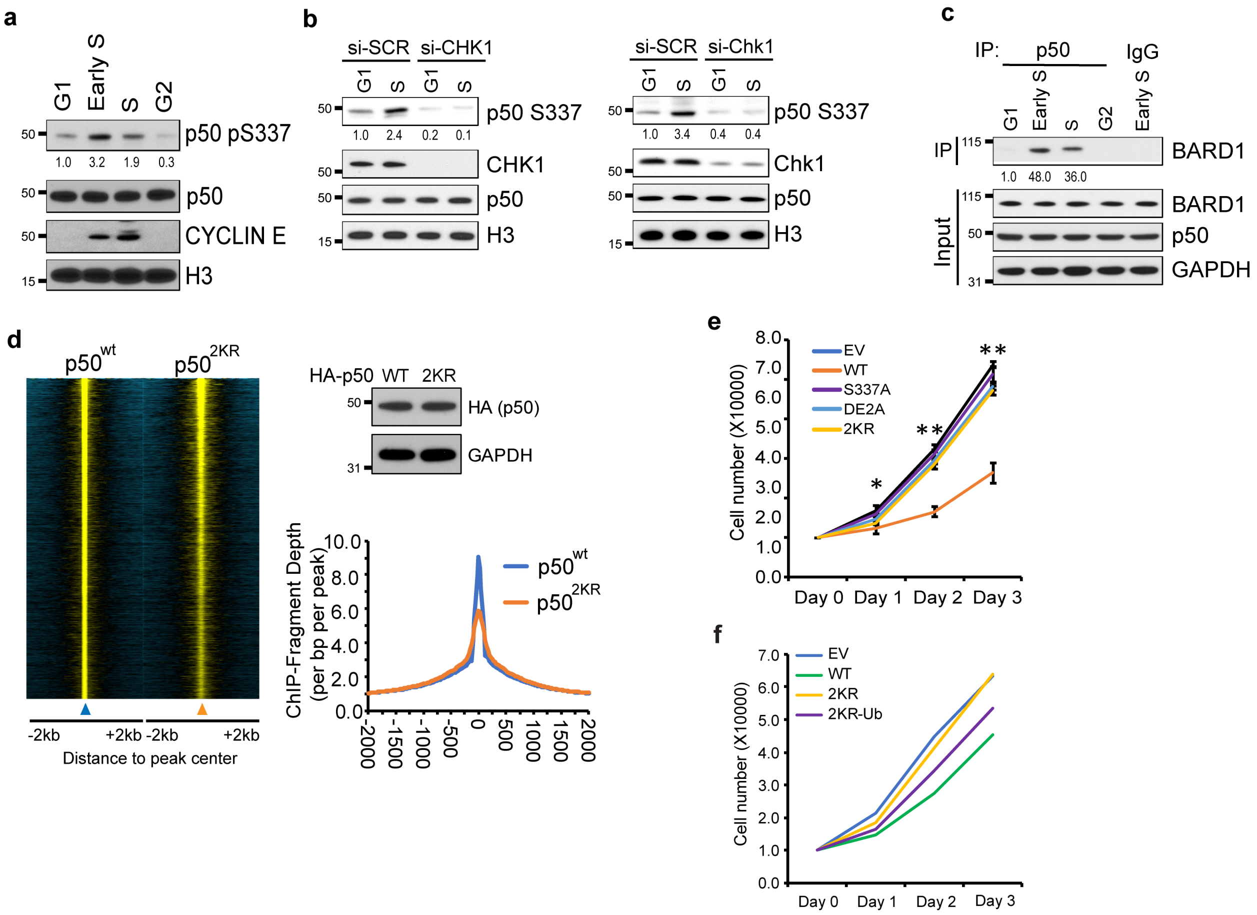

Supplementary Fig. 5. p50 mono-ubiquitination regulates cell cycle progression. (**a**) 293T cells synchronized by double-thymidine block were harvested at the indicated cell cycle stage and nuclear fractions isolated. IB using anti-p50-phospho-S337, anti-p50 and anti-Cyclin E performed. **(b)** 293T cells (left) or WT MEFs (right) transfected with the indicated siRNA were synchronized by double-thymidine block and harvested at the indicated cell cycle stage. Nuclear fractions were isolated and IB using anti-p50-phospho-S337 or the indicated antibody performed. (**c**) Co-IP in 293T cells synchronized and released as in **a**. IP with anti-p50 or IgG control and IB with anti-BARD1 or indicated antibody. (**d**) ChIP-seq analysis of p50 binding peaks. Heatmap (left) and histogram (right lower) for average peak intensity was plotted for ChIP-Seq data from cells expressing $p50^{wt}$ or $p50^{2KR}$. IB (right upper) demonstrates equal HA-p50 expression. (**e**) 293T cells stably expressing empty vector (EV) or the indicated p50 mutant were plated and cell number counted every 24 hrs. Data show mean number from three biologically independent experiments, ± SEM. **P* = 0.015, ***P* < 0.0001, EV vs. WT, two-sided unpaired Student *t* test. (**f**) *Nfkb1*$^{-/-}$ MEFs expressing EV, $p50^{wt}$, $p50^{2KR}$ or $p50^{2KR\text{-}Ub}$ (2KR-Ub) were plated and cell number counted every 24 hrs. Data show mean number from two biologically independent experiments. All blots are representative of at least two biologically independent experiments. Analysis of fold-change normalized to control lane shown below IB where indicated.

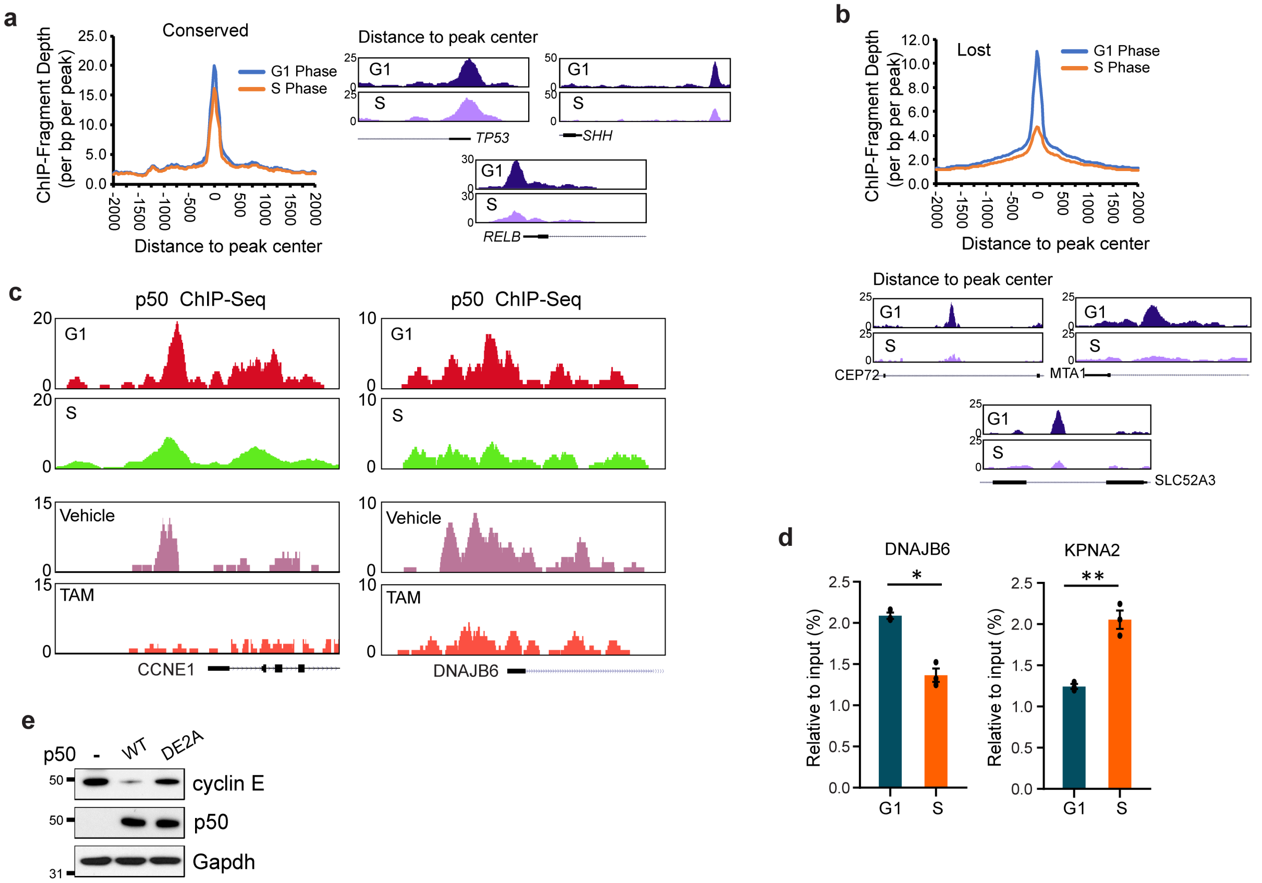

Supplementary Fig. 6. p50 PTM regulates Cyclin E expression
(**a**) Histogram (left) of average peak intensity of p50 binding peaks that were present in G1 and retained in S phase. Schematic of three p50 binding peaks in this group (*TP53*, *SHH* and *RELB*, right) during G1 and S phase at the indicated locus promoter/enhancer. (**b**) Histogram (upper) of average peak intensity of p50 binding peaks that were lost in S phase compared to G1. Schematic of three p50 binding peaks in this group (*CEP72*, *MTA1* and *SLC52A3*, lower) during G1 and S phase at the indicated locus promoter/enhancer. (**c**) Schematic of the p50 binding peak at the *CCNE1* (left) and *DNAJB6* (right) promoter/enhancer during G1 and S phase (upper), and in TopBP1^{ER} cells treated with either vehicle or TAM (lower). (**d**) ChIP-qPCR of p50 enrichment at the indicated promoter in 293T cells at G1 and S phase. Data show mean enrichment of p50 relative to input and IgG control, ± SEM from 3 biologically independent experiments. *P = 0.038, **P = 0.014, two-sided unpaired Student t test. (**e**) Tumors from immortal *Nfkb1*$^{-/-}$ MEFs stably expressing the indicated construct were harvested and IB with the indicated antibody performed. Blot is representative of at least two biologically independent experiments.

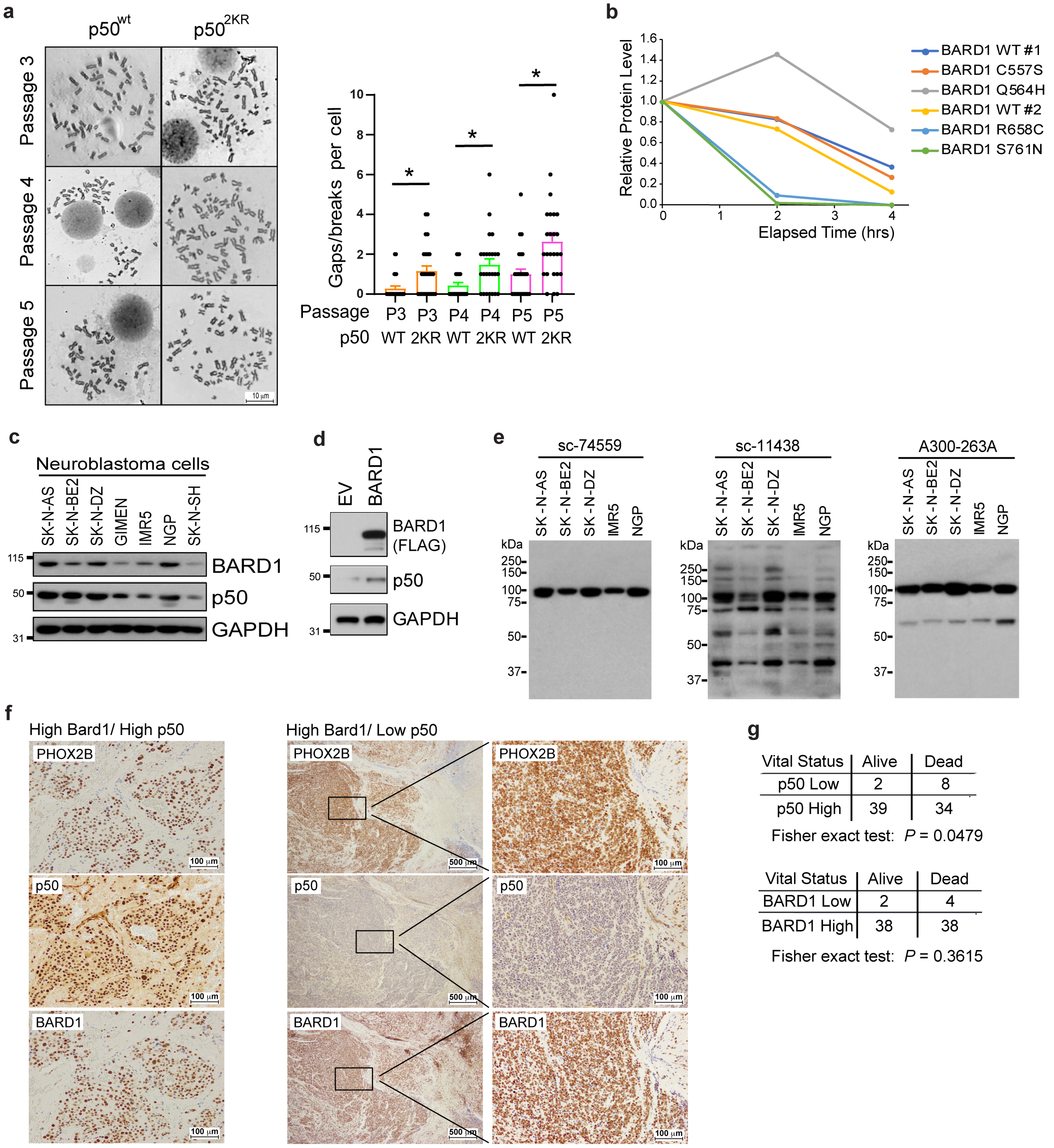

Supplementary Fig. 7. BARD1 and p50 protein in human cancer. (**a**) Analysis of spontaneous breaks and gaps in primary *Nfkb1*$^{-/-}$ MEFs expressing either p50^{wt} or p50^{2KR}. Cells were continuously passaged and metaphase spreads analyzed at the indicated passage. Data show gaps or breaks from 25 random metaphase cells, ± SEM *P < 0.005, two-sided unpaired Student *t* test. Representative metaphase spreads from each passage shown (left). (**b**) Quantification of data from Fig. 7c indicates amount of

p50 protein in the presence of WT BARD1 or the indicated BARD1 mutant. (**c**) Neuroblastoma cell lines were grown and lysates analyzed by IB with anti-BARD1 (sc-74559) or anti-p50 antibody. (**d**) GIMEN neuroblastoma cells were infected with lentivirus expressing empty vector (EV) or FLAG-BARD1 and IB performed with anti-FLAG or anti-p50 antibody. (**e**) Immunoblots in neuroblastoma cell lines using the indicated anti-BARD1 antibody. Left, mouse monoclonal antibody (sc-74559, 1:1000). Center, rabbit polyclonal antibody (sc-11438, 1:500). Right, rabbit polyclonal antibody (A300-263A,1:2500). (**f**) IHC staining of nuclear p50 and BARD1 in neuroblastoma samples. Only PHOX2B positive cells scored as positive or negative. High BARD1/ High p50 (left, 20x magnification). High BARD1/ Low p50 (center and right). Right-sided images show magnification (20x) of boxed area. (**g**) Data show numbers of breast cancer patients in each indicated survival category analyzed by one-sided Fisher's exact test. All blots are representative of at least two biologically independent experiments.

Supplementary Tables

Supplementary Table 1. List of proteins that interact with HA-p50 identified by LC-MS/MS.

Protein	Description	Score
RELA	RELA proto-oncogene, NF-kB subunit	104
PARP6	Poly (ADP-ribose) polymerase family member 6	74
SALL4B	Sal-like protein 4	52
UBAP1	Ubiquitin associated protein 1	48
PRMT5	Protein arginine methyltransferase 5	48
KEAP1	Kelch-like ECH-associated protein 1	45
BARD1	BRCA1 associated RING domain 1	43

Supplementary Table 2. List of oligonucleotides used and their applications.

Oligo Name	Sequence	Application	Supplier	Cat. No.
KPNA2 ChIP F1	CAC ACG GTC TTT GAG CTG AG	ChIP-qPCR	IDT	NA
KPNA2 ChIP R1	TGA GTC TGT ACC TGC GAA GC	ChIP-qPCR	IDT	NA
DNAJB6 ChIP F1	TCC CAA AGT GCT GGG ATT AC	ChIP-qPCR	IDT	NA
DNAJB6 ChIP R1	GGT TAT CCC CTC CTG AGA GC	ChIP-qPCR	IDT	NA
CCNE1 ChIP F1	GGC CTG AAG CCT TGG TTC TA	ChIP-qPCR	IDT	NA
CCNE1 ChIP R1	CGG GTG GAA TGT AAA CAC G	ChIP-qPCR	IDT	NA
IgK WT κB site	TCGAGTTAGATGGGGACTTTCC AGGCAC	EMSA	IDT	NA
IgK -1A κB site	TCGAGTTAGATGGGGAATTTCC AGGCAC	EMSA	IDT	NA
CD44 κB site	TCGAGTTAGATGGGGATCCTCC AGGCAC	EMSA	IDT	NA
TNFα κB site	TCGAGTTAGATGGGGGCTTTCC AGGCAC	EMSA	IDT	NA
H2B κB site	TCGAGTTAGATGGGGGATTCCC AGGCAC	EMSA	IDT	NA
Non-Specific competitor	TCGAGTTAGATGTAACCGACTA GGCAC	EMSA	IDT	NA
siRNA scrambled control	CCUACGCCACCAAUUUCGUUU	siRNA Knockdown	IDT	NA
siGenome Smart Pool targeting BARD1 #1	GAGCACAUCUUCUGUAGUA	siRNA Knockdown	Dharmacon	D-003873-01
siGenome Smart Pool targeting BARD1 #2	GUAGUAAGCUUCGAAAUUU	siRNA Knockdown	Dharmacon	D-003873-02
siGenome Smart Pool targeting BARD1 #3	CAAAGUACCUUGAAGUGUA	siRNA Knockdown	Dharmacon	D-003873-03
siGenome Smart Pool targeting BARD1 #4	GAAAUAGACUUACUAGCAA	siRNA Knockdown	Dharmacon	D-003873-04
siGenome Smart Pool targeting CHEK1 #1	GCAACAGUAUUUCGGUAUA	siRNA Knockdown	Dharmacon	D-003255-06
siGenome Smart Pool targeting CHEK1 #2	GGACUUCUCUCCAGUAAAC	siRNA Knockdown	Dharmacon	D-003255-07
siGenome Smart Pool targeting CHEK1 #3	AAAGAUAGAUGGUACAACA	siRNA Knockdown	Dharmacon	D-003255-08
siGenome Smart Pool targeting CHEK1 #4	CCACAUGUCCUGAUCAUAU	siRNA Knockdown	Dharmacon	D-003255-09
shRNA scrambled control	AAGCGCGCTTTGTAGGATTCG	shRNA Knockdown	IDT	NA

shRNA targeting sequence: BARD1 #1	AAGATAAACCTAGGAAAAGTT	shRNA Knockdown	IDT	NA
shRNA targeting sequence: BARD1 #2	AAGTGGCTCCTTGACAGAATC	shRNA Knockdown	IDT	NA
shRNA targeting sequence: BCL3 #1	AAGCACATGCACCTACCCATA	shRNA Knockdown	IDT	NA
shRNA targeting sequence: BCL3 #2	AATGCCACCCACATCTTCCAT	shRNA Knockdown	IDT	NA
