# Supplementary material for: p50 mono-ubiquitination and interaction with BARD1 regulates cell cycle progression and maintains genome stability
Source: Nat Commun. 2020 Oct 6;11:5007. doi: 10.1038/s41467-020-18838-2 (PMC7538584; doi:10.1038/s41467-020-18838-2)
Supplement: Supplementary file 3 — Description of Additional Supplementary Files [file 41467_2020_18838_MOESM3_ESM.pdf]

Description of Additional Supplementary Files

File Name: Supplementary Data 1
Description: ChIP-Seq data showing location of chromatin peaks bound by endogenous p50 in 293T cells expressing TopBP1ER treated with either vehicle or tamoxifen (TAM).

File Name: Supplementary Data 2
Description: ChIP-Seq data showing location of chromatin peaks bound by HAp50WT or HA-p502KR.

File Name: Supplementary Data 3
Description: List of periodically expressed genes in Cyclebase 3.0. Overlap between Cyclebase 3.0 periodic genes and genes identified in ChIP-Seq experiments using cells expressing TopBP1ER treated with vehicle and TAM or cells isolated at G1 and S phase. List of genes from each subset of the Venn diagram shown in Fig 6i.

File Name: Supplementary Data 4
Description: ChIP-Seq data showing location of chromatin peaks bound by endogenous p50 in synchronized cells harvested at either G1 or S Phase.
