# Supplementary material for: p50 mono-ubiquitination and interaction with BARD1 regulates cell cycle progression and maintains genome stability
Source: Nat Commun. 2020 Oct 6;11:5007. doi: 10.1038/s41467-020-18838-2 (PMC7538584; doi:10.1038/s41467-020-18838-2)
Supplement: Supplementary file 8 — Reporting Summary [file 41467_2020_18838_MOESM8_ESM.pdf]

nature research

Corresponding author(s): Bakhtiar Yamini

Last updated by author(s): Aug 18, 2020

Reporting Summary

Nature Research wishes to improve the reproducibility of the work that we publish. This form provides structure for consistency and transparency in reporting. For further information on Nature Research policies, see our Editorial Policies and the Editorial Policy Checklist.

Statistics

For all statistical analyses, confirm that the following items are present in the figure legend, table legend, main text, or Methods section.

n/a	Confirmed	
☐	☒	The exact sample size (*n*) for each experimental group/condition, given as a discrete number and unit of measurement
☐	☒	A statement on whether measurements were taken from distinct samples or whether the same sample was measured repeatedly
☐	☒	The statistical test(s) used AND whether they are one- or two-sided<br>*Only common tests should be described solely by name; describe more complex techniques in the Methods section.*
☒	☐	A description of all covariates tested
☒	☐	A description of any assumptions or corrections, such as tests of normality and adjustment for multiple comparisons
☐	☒	A full description of the statistical parameters including central tendency (e.g. means) or other basic estimates (e.g. regression coefficient) AND variation (e.g. standard deviation) or associated estimates of uncertainty (e.g. confidence intervals)
☐	☒	For null hypothesis testing, the test statistic (e.g. *F*, *t*, *r*) with confidence intervals, effect sizes, degrees of freedom and *P* value noted<br>*Give P values as exact values whenever suitable.*
☒	☐	For Bayesian analysis, information on the choice of priors and Markov chain Monte Carlo settings
☒	☐	For hierarchical and complex designs, identification of the appropriate level for tests and full reporting of outcomes
☒	☐	Estimates of effect sizes (e.g. Cohen's *d*, Pearson's *r*), indicating how they were calculated

Our web collection on statistics for biologists contains articles on many of the points above.

Software and code

Policy information about availability of computer code

Data collection	Illumina HiSeq4000 platform<br>Biorad IQ5 Real-Time PCR Detection System
Data analysis	Single end 50bp short reads were retrieved from the Genomics Core Facility. Samples were first trimmed with Trimmomatic (v0.38) to remove low quality reads. Subsequently, reads generated from PCR amplification were filtered with Broad Institute Picard Tools (v 2.18.27). The short reads were mapped to human genome (hg38) with Bowtie2 (v2.3.4.3), and sorted with Samtools (v1.9). Homer pipeline (v4.10) was used for peak calling (findPeaks) and annotation (annotatePeaks.pl). the false discovery rate (FDR) was set as default (0.001). ImageJ (v1.52) bundled with 64-bit Java 1.8.0_112 was used to make quantitative analysis of western blot results.<br>GraphPad Prism 7 was used to generated bar graph and dot plot, as well as the fitting curve for protein stability assays.

For manuscripts utilizing custom algorithms or software that are central to the research but not yet described in published literature, software must be made available to editors and reviewers. We strongly encourage code deposition in a community repository (e.g. GitHub). See the Nature Research guidelines for submitting code & software for further information.

Data

Policy information about availability of data

All manuscripts must include a data availability statement. This statement should provide the following information, where applicable:

- Accession codes, unique identifiers, or web links for publicly available datasets
- A list of figures that have associated raw data
- A description of any restrictions on data availability

ChIP-Seq data have been deposited at the NCBI GEO repository under accession number: GSE129618 https://www.ncbi.nlm.nih.gov/geo/query/acc.cgi?acc=GSE129618. Source data are provided with this paper. All other data can be provided by the corresponding author upon reasonable request.

Figure 5 and 6 have associated raw data.

Field-specific reporting

Please select the one below that is the best fit for your research. If you are not sure, read the appropriate sections before making your selection.

- [x] Life sciences
- [ ] Behavioural & social sciences
- [ ] Ecological, evolutionary & environmental sciences

For a reference copy of the document with all sections, see nature.com/documents/nr-reporting-summary-flat.pdf

Life sciences study design

All studies must disclose on these points even when the disclosure is negative.

Sample size	There was no specific statistical analysis used to determine sample size. For breast cancer and neuroblastoma, total available numbers of specimens were used. For in vitro experiments, we used 3 experimental replicates that demonstrated reproducible results. We used n= 5 for murine tumors.
Data exclusions	For analysis of Breast cancer survival, 2 patients had data excluded because of unknown date of diagnosis.
Replication	All experimental findings have been verified by two to three biological replications. Representative blots are shown. The results are reproducible. All quantifications and semi-quantitative analyses are representative of at least two and usually three independent biological experiments.
Randomization	Randomization was not relevant to our human tumors as this involved retrospective analysis of all available tumors. For murine tumors, mice were randomly recruited prior to use.
Blinding	IHC grading was performed in a blinded fashion by two independent observers. For other studies, investigators were blinded to group allocation during data collection and analysis

Reporting for specific materials, systems and methods

We require information from authors about some types of materials, experimental systems and methods used in many studies. Here, indicate whether each material, system or method listed is relevant to your study. If you are not sure if a list item applies to your research, read the appropriate section before selecting a response.

Materials & experimental systems

n/a	Involved in the study	
☐	☒	Antibodies
☐	☒	Eukaryotic cell lines
☒	☐	Palaeontology and archaeology
☐	☒	Animals and other organisms
☐	☒	Human research participants
☒	☐	Clinical data
☒	☐	Dual use research of concern

Methods

n/a	Involved in the study	
☐	☒	ChIP-seq
☒	☐	Flow cytometry
☒	☐	MRI-based neuroimaging

Antibodies

Antibodies used

Mouse monoclonal FLAG M2 antibody (Sigma-Aldrich, Cat. No. F1804, RRID:AB_262044)
Rabbit monoclonal FLAG antibody (Cell Signaling, Cat. No. #14793, RRID:AB_2572291)
Mouse monoclonal BARD1 (E-11) antibody (Santa Cruz, Cat. No. sc-74559, RRID:AB_2061237)
Rabbit polyclonal BARD1 antibody (Bethyl Laboratories, Cat. No. A300-263A, RRID:AB_2061250)
Rabbit polyclonal BARD1 (H-300) antibody (Santa Cruz, Cat. No. sc-11438, RRID:AB_2061240)
Mouse monoclonal NFκB1/p50 (E-10) antibody (Santa Cruz, Cat. No. sc-8414X, RRID:AB_628015)
Rabbit polyclonal NFκB1/p50 (H-119) antibody (Santa Cruz, Cat. No. sc-7178X, RRID:AB_650211)
Rabbit monoclonal NFκB1/p50 (E381) antibody (Abcam, Cat. No. ab32360, RRID: AB_776748)
Rabbit polyclonal Anti-HA tag antibody - ChIP Grade (Abcam, Cat. No. Ab9110, RRID:AB_307019)
Normal rabbit IgG (Santa Cruz, Cat. No. sc-2027, RRID:AB_737197)
Normal mouse IgG (Santa Cruz, Cat. No. sc-2025, RRID:AB_737182)
Mouse monoclonal purified anti-S-Tag Antibody (BioLegend, Cat. No. 688102, RRID:AB_2629593)
Mouse monoclonal Chk1 (G-4) antibody (Santa Cruz, Cat. No. sc-8408, RRID:AB_627257)
Mouse monoclonal GAPDH (6C5) antibody (Santa Cruz, Cat. No. sc-32233, RRID:AB_627679
Mouse monoclonal Myc tag (9E10) antibody (Santa Cruz, Cat. No. sc-40, RRID:AB_627268
Mouse monoclonal BRCA1 (D-9) antibody (Santa Cruz, Cat. No. sc-6954, RRID:AB_626761

Rabbit polyclonal cyclin E (C-19) antibody (Santa Cruz, Cat. No. sc-198, RRID:AB_631346
Rabbit polyclonal Bcl-3 (C-14) antibody (Santa Cruz, Cat. No. sc-185, RRID:AB_2258970
Rabbit monoclonal PHOX2B antibody [EPR14423] (Abcam, Cat. No. ab183741, RRIS: Not Available)
Mouse monoclonal Histone H3 (1G1) antibody (Santa Cruz, Cat. No. sc-517576, RRID: Not Available)
Rabbit polyclonal NFκB1/p50 S337 phosphorylation antibody (Custom Antibody made by YenZym)
Donkey anti-Mouse IgG (H+L) Highly Cross-Adsorbed Secondary Antibody, HRP (ThermoFisher Scientific, A16017)
Donkey anti-Rabbit IgG (H+L) Highly Cross-Adsorbed Secondary Antibody, HRP (ThermoFisher Scientific, A16035)

Validation

Mouse monoclonal FLAG M2 antibody (Sigma-Aldrich, Cat. No. F1804, RRID:AB_262044)
Specificity & Sensitivity provided by company, the antibody was validated with: Immunofluorescence analysis in MDCK canine kidney epithelial cells, transfected with FLAG tagged myr-PKCz are labeled with Monoclonal ANTI-FLAG M2 antibody.
Rabbit monoclonal FLAG antibody (Cell Signaling, Cat. No. #14793, RRID:AB_2572291)
Specificity & Sensitivity provided by company, the antibody was validated with: Western blot analysis of extracts from 293T cells, mock transfected (-) or transfected with DYKDDDDK-GFP (N-terminal DDK-Tag; +), GFP-DYKDDDDK (C-terminal DDK-Tag; +), human CASQ1-DYKDDDDK (C-terminal DDK-Tag; +), or human FoxG1-DYKDDDDK (C-terminal DDK-Tag; +) as indicated, using DYKDDDDK Tag (D6W5B) Rabbit mAb.
Mouse monoclonal BARD1 (E-11) antibody (Santa Cruz, Cat. No. sc-74559, RRID:AB_2061237)
Specificity & Sensitivity provided by company, the antibody was validated with: Western blot analysis of BARD1 expression in U-2 OS (A), BT-20 (B) and MCF7 (C) whole cell lysates. Immunoperoxidase staining of formalin fixed, paraffin-embedded human testis tissue showing nuclear staining of cells in seminiferous ducts and nuclear and cytoplasmic staining of Leydig cells. Immunofluorescence staining of formalin-fixed HeLa cells showing nuclear and cytoplasmic localization.
Rabbit polyclonal BARD1 antibody (Bethyl Laboratories, Cat. No. A300-263A, RRID:AB_2061250)
Specificity & Sensitivity provided by company, the antibody was validated with: Western blot with whole cell lysate from Jurkat, Hep-G2, K-562, MCF-7, and A-549 cells prepared using NETN lysis buffer. Antibody: Affinity purified rabbit anti-BARD1 antibody A300-263A used for WB at 0.04 μg/ml. Detection: Chemiluminescence with an exposure time of 30 seconds. It is also validated with Western blot of immunoprecipitates. Whole cell lysate (1.0 mg per IP reaction; 20% of IP loaded) from MCF-7 cells prepared using NETN lysis buffer. Antibodies: Affinity purified rabbit anti-BARD1 antibody A300-263A used for IP at 6 μg per reaction. BARD1 was also immunoprecipitated by a previous lot of this antibody. For blotting immunoprecipitated BARD1, A300-263A was used at 0.04 μg/ml. Detection: Chemiluminescence with an exposure time of 10 seconds.
Rabbit polyclonal BARD1 (H-300) antibody (Santa Cruz, Cat. No. sc-11438, RRID:AB_2061240)
Specificity & Sensitivity provided by company, the antibody was validated with: Western blot analysis of BARD1 expression in U-2 OS whole cell lysate. It is also validated by publications with WB on NuTu-19, HEK293T, TAC-2, MCF-7, and DR-U2OS.
Mouse monoclonal NFκB1/p50 (E-10) antibody (Santa Cruz, Cat. No. sc-8414X, RRID:AB_628015)
Specificity & Sensitivity provided by company, the antibody was validated with: Immunoperoxidase staining of formalin fixed, paraffin-embedded human fallopian tube tissue showing cytoplasmic staining of glandular cells. Western blot analysis of NFκB p50 expression in A431 (A), THP-1 (B) and Daudi (C) whole cell lysates. Intracellular FCM analysis of fixed and permeabilized Jurkat cells.
Rabbit polyclonal NFκB1/p50 (H-119) antibody (Santa Cruz, Cat. No. sc-7178X, RRID:AB_650211)
Specificity & Sensitivity provided by company, the antibody was validated with: Western blot analysis of NFκB p50 and p105 expression in A-431 whole cell lysate. Immunofluorescence staining of methanol-fixed A-431 cells showing cytoplasmic and nuclear staining.
Rabbit monoclonal NFκB1/p50 (E381) antibody (Abcam, Cat. No. ab32360, RRID: AB_776748)
Specificity & Sensitivity provided by company, the antibody was validated with: Western blot was shown to specifically react with NFkB p105 / p50 when NFkB p105 / p50 knockout samples were used. Wild-type and NFkB p105 / p50 knockout samples were subjected to SDS-PAGE.
Rabbit polyclonal Anti-HA tag antibody - ChIP Grade (Abcam, Cat. No. Ab9110, RRID:AB_307019)
Specificity & Sensitivity provided by company, the antibody was validated with: Staining HA-tagged proteins in HeLa cells by ICC/IF. Immunoprecipitation with a nuclear lysate of HEK293T cells transiently expressing HA-tagged protein and followed by Western blot. ChIP analysis with mouse embryonic stem whole cell lysate treated with disuccinimidyl glutarate (cross-linking agent). The bound DNA was quantitated by real-time PCR.
Normal rabbit IgG (Santa Cruz, Cat. No. sc-2027, RRID:AB_737197)
Specificity & Sensitivity provided by company, the antibody was validated with: It was used as negative control for Western blotting, immunoprecipitation and immunohistochemistry applications, and cited with more than 2000 publications.
Normal mouse IgG (Santa Cruz, Cat. No. sc-2025, RRID:AB_737182)
Specificity & Sensitivity provided by company, the antibody was validated with: It was used as negative control for Western blotting, immunoprecipitation and immunohistochemistry applications, and cited with more than 2000 publications.
Mouse monoclonal purified anti-S-Tag Antibody (BioLegend, Cat. No. 688102, RRID:AB_2629593)
Specificity & Sensitivity provided by company, the antibody was validated with: Recombinant protein with S-tag was resolved by electrophoresis, transferred to nitrocellulose, and probed with purified anti-S-Tag (clone BL26881) antibody. Proteins were visualized using a goat anti-mouse-IgG secondary antibody conjugated to HRP and chemiluminescence detection.
Mouse monoclonal Chk1 (G-4) antibody (Santa Cruz, Cat. No. sc-8408, RRID:AB_627257)
Specificity & Sensitivity provided by company, the antibody was validated with: Western blot analysis of Chk1 expression in MDA-MB-231, C6, Jurkat, MEG-01, HEL 92.1.7 and MOLT-4 whole cell lysates. Immunoperoxidase staining of formalin fixed, paraffin-embedded human testis tissue showing nuclear and cytoplasmic staining of cells in seminiferous ducts and Leydig cells.
Mouse monoclonal GAPDH (6C5) antibody (Santa Cruz, Cat. No. sc-32233, RRID:AB_627679
Specificity & Sensitivity provided by company, the antibody was validated with: Immunofluorescence staining of methanol-fixed KNRK cells showing cytoplasmic localization. Western blot analysis of GAPDH expression in non-transfected 293T: sc-117752, human GAPDH transfected 293T: sc-113612 and A549 whole cell lysates.
Mouse monoclonal Myc tag (9E10) antibody (Santa Cruz, Cat. No. sc-40, RRID:AB_627268
Specificity & Sensitivity provided by company, the antibody was validated with: Direct near-infrared western blot analysis of c-Myc expression in COS whole cell lysates prepared from non-transfected cells and c-Myc fusion protein transfected cells. Immunofluorescence staining of methanol-fixed COS cells transfected with c-Myc fusion protein showing cytoplasmic staining.
Mouse monoclonal BRCA1 (D-9) antibody (Santa Cruz, Cat. No. sc-6954, RRID:AB_626761

Specificity & Sensitivity provided by company, the antibody was validated with: Western blot analysis of BRCA1 expression in A-431, HeLa and MCF7 nuclear extracts. Immunofluorescence staining of formalin-fixed, UVA laser-microirradiated HeLa cells showing nuclear staining of cells with DNA damage.
Rabbit polyclonal cyclin E (C-19) antibody (Santa Cruz, Cat. No. sc-198, RRID:AB_631346
Specificity & Sensitivity provided by company, the antibody was validated with: Immunoperoxidase staining of formalin-fixed, paraffin-embedded human breast carcinoma tissue showing nuclear staining. Western blot analysis of cyclin E expression in non-transfected: sc-117752 and human cyclin E transfected: sc-170464 293T whole cell lysates.
Rabbit polyclonal Bcl-3 (C-14) antibody (Santa Cruz, Cat. No. sc-185, RRID:AB_2258970
Specificity & Sensitivity provided by company, the antibody was validated with: Western blot analysis of Bcl-3 expression in NAMALWA and WEHI-3 whole cell lysates. Immunoperoxidase staining of formalin fixed, paraffin-embedded human colon tissue showing cytoplasmic and nuclear staining of BCL3 in glandular cells.
Rabbit monoclonal PHOX2B antibody [EPR14423] (Abcam, Cat. No. ab183741, RRIS: Not Available)
Specificity & Sensitivity provided by company, the antibody was validated with: Immunohistochemical analysis of paraffin-embedded Human neuroblastoma tissue labeling PHOX2B with ab183741 at 1/1000 dilution. Western blot analysis with Neuro-2a and SH-SY5Y cell lysate.
Mouse monoclonal Histone H3 (1G1) antibody (Santa Cruz, Cat. No. sc-517576, RRID: Not Available)
Specificity & Sensitivity provided by company, the antibody was validated with: Detection of Histone H3 of mouse, rat and human origin by WB and IF.
Rabbit polyclonal NFκB1/p50 S337 phosphorylation antibody (Custom Antibody made by YenZym)
Specificity & Sensitivity provided in this paper, the antibody was validated with: Western blot of phosphorylated protein with specific peptide and competitive peptide, as well as p50 mutant, S337A.

Eukaryotic cell lines

Policy information about cell lines

Cell line source(s)	HEK293T, HeLa, and MCF7 cell lines were obtained from ATCC. Human neuroblastoma cell lines (SK-N-AS, NBL-S, SHEP, SK-NDZ, SK-N-BE2, GIMEN, IMR5, NGP, SK-N-SH, SH-SY5Y, NBL-WN) were a gift from Dr. S Cohn (The University of Chicago). The BRCA1 mutant cell line HCC1937 was a gift from Dr. P Connell (The University of Chicago). Bard-null mouse mammary carcinoma cell line (10-05) was a gift from Dr. R Baer (Columbia University Medical Center). Primary MEFs were freshly isolated from day 13 embryonic mice in our lab according to standard protocol as described in Method section. Immortal Nfkb1-/- MEFs were originally obtained from Dr. XY Fu at the University of Chicago.
Authentication	Primary MEFs were from our lab and freshly isolated from our animal colony. BRCA1 mutant and Bard1-null cells were authenticated by immunoblot. The other cells lines used were not authenticated in our lab.
Mycoplasma contamination	Cells were routinely screened for free of Mycoplasma contaminations. All cell lines are Mycoplasma negative with this study.
Commonly misidentified lines (See ICLAC register)	None of the cell lines used in this study is commonly misidentified cell line.

Animals and other organisms

Policy information about studies involving animals; ARRIVE guidelines recommended for reporting animal research

Laboratory animals	Six- to 7-week-old male nude mice (Hsd:Athymic Nude-Foxn1nu) purchased from Harlan-Envigo. Animals were maintained in a specific pathogen-free environment with 12 hr light/dark cycle, 40% humidity 72 degree F and food/water ad lib.
Wild animals	This study did not involve wild animals.
Field-collected samples	This study did not involve samples collected from field.
Ethics oversight	Relevant experiments were approved by Institutional Review Board (IRB) of University of Chicago.

Note that full information on the approval of the study protocol must also be provided in the manuscript.

Human research participants

Policy information about studies involving human research participants

Population characteristics	For neuroblastoma, patients of any age with tissue available were included. For breast cancer, patients who had tissue on the TMAs were included. Populations for both groups were wildly mixed. No link to patient identifying characteristics is publically available.
Recruitment	No specific recruitment was used. For neuroblastoma, patients with an adequate amount of availible archived tissue specimen were incorporated. For breast cancer, patients were included if their sample had been included on the TMA. Self-selection bias was not involved as patient samples were included if they had tissue in the archive. For neuroblastoma, bias may have occurred in that only patients with adequate tissue sample were included in the study. However, this would not have affected the results and the study only examined the correlation between the staining of two proteins in the samples.
Ethics oversight	The University of Chicago IRB

Note that full information on the approval of the study protocol must also be provided in the manuscript.

ChIP-seq

Data deposition

☒ Confirm that both raw and final processed data have been deposited in a public database such as GEO.

☒ Confirm that you have deposited or provided access to graph files (e.g. BED files) for the called peaks.

Data access links<br>*May remain private before publication.*	https://www.ncbi.nlm.nih.gov/geo/query/acc.cgi?acc=GSE129618
Files in database submission	GSM3717125 for Input DNA as background control<br>GSM3717126 for NFKB1/p50 ChIP-Seq at G1<br>GSM3717127 for NFKB1/p50 ChIP-Seq at S<br>GSM3717128 for NFKB1/p50 ChIP-Seq in cells treated with vehicle<br>GSM3717129 for NFKB1/p50 ChIP-Seq in cells treated with Tamoxifen<br>GSM3717130 for NFKB1/p50 ChIP-Seq in cells expressing HA-p50 WT<br>GSM3717131 for NFKB1/p50 ChIP-Seq in cells expressing HA-p50 2KR mutant
Genome browser session<br>(e.g. UCSC)	No longer applicable

Methodology

Replicates	No replicates
Sequencing depth	Single end 50bp short reads were retrieved from the Genomics Core Facility (University of Chicago). For p50 ChIP-Seq at G1, 33.4M reads were sequenced, and 23M reads were uniquely mapped to hg38. For p50 ChIP-Seq at S, 33M reads were sequenced, and 22.7M reads were uniquely mapped to hg38. For p50 ChIP-Seq in cells treated with vehicle, 33.7M reads were sequenced, and 21.9M reads were uniquely mapped to hg38. For p50 ChIP-Seq in cells treated with tamoxifen, 37.5M reads were sequenced, and 21.3M reads were uniquely mapped to hg38. For HA ChIP-Seq in cells with HA-p50 WT expression, 33.4 reads were sequenced, and 22.9M reads were uniquely mapped to hg38. For HA ChIP-Seq in cells with HAp50 mutant expression, 31.6M reads were sequenced, and 21.8M reads were uniquely mapped to hg38.
Antibodies	Rabbit polyclonal NFκB1/p50 (H-119) antibody (Santa Cruz, Cat. No. sc-7178X) was used for endogenous p50 ChIP-Seq.<br>Rabbit polyclonal Anti-HA tag antibody - ChIP Grade (Abcam, Cat. No. Ab9110) was used for HA-tagged p50 ChIP-Seq.
Peak calling parameters	All peak calling was performed with Homer pipeline (v4.10), and the default parameters were used without modification, the default false discovery rate (FDR) for Homer software was 0.001.
Data quality	We assessed the quality of the sequencing reads using FastQC. Homer pipeline (v4.10) was used for peak calling (findPeaks) and annotation (annotatePeaks.pl). the false discovery rate (FDR) was set as default (0.001). For p50 ChIP-Seq at G1, 1212 peaks were identified. For p50 ChIP-Seq at S, 611 peaks were identified. For p50 ChIP-Seq in cells treated with vehicle, 705 peaks were identified. For p50 ChIP-Seq in cells treated with tamoxifen, 402 peaks were identified. For HA ChIP-Seq in cells with HA-p50 WT expression, 5110 peaks were identified, and for HA ChIP-Seq in cells with HA-p50 mutant expression, 5535 peaks were identified.
Software	Single end 50bp short reads were retrieved from the Genomics Core Facility. Samples were first trimmed with Trimmomatic (v0.38) to remove low quality reads. Subsequently, reads generated from PCR amplification were filtered with Broad Institute Picard Tools (v 2.18.27). The short reads were mapped to human genome (hg38) with Bowtie2 (v2.3.4.3), and sorted with Samtools (v1.9). Homer pipeline (v4.10) was used for peak calling (findPeaks) and annotation (annotatePeaks.pl). the false discovery rate (FDR) was set as default (0.001). The quantification of co-immunoprecipitation, protein stability, and western blot results was performed with ImageJ (v1.52a), gel analysis tool set. Protein stability, ubiquitination kinetics model and half-life were fit and calculated with Prism Graphpad (v7).
